# ‘If he sees it with his own eyes, he will understand’: how gender informed the content and delivery of a maternal nutrition intervention in Burkina Faso

**DOI:** 10.1093/heapol/czaa012

**Published:** 2020-02-27

**Authors:** Jasmin Isler, N Hélène Sawadogo, Guy Harling, Till Bärnighausen, Maya Adam, Ali Sié, Shannon A McMahon

**Affiliations:** c1 Institute of Global Health, Heidelberg University, Im Neuenheimer Feld 130/3, 69120 Heidelberg, Germany; c2 Nouna Health Research Center, Rue Namory Kéita, Nouna, Burkina Faso; c3 Institute for Global Health, University College London, Mortimer Market Centre, off Capper Street, London WC1E 6JB, UK; c4 Department of Epidemiology, Harvard T.H. Chan School of Public Health, 677 Huntington Ave, Boston, MA 02115, USA; c5 Harvard Center for Population and Development Studies, Harvard University, 9 Bow Street, Cambridge, MA 02138, USA; c6 Africa Health Research Institute, KwaZulu-Natal, South Africa; c7 MRC/Wits Rural Public Health and Health Transitions Research Unit (Agincourt), University of the Witwatersrand, 1 Jan Smuts Avenue, Braamfontein 2000, Johannesburg, South Africa; c8 Department of Global Health and Population, Harvard T.H. Chan School of Public Health, 677 Huntington Ave, Boston, MA 02115, USA; c9 Stanford Center for Health Education, Stanford School of Medicine, Stanford University, 450 Serra Mall, Stanford, CA 94305, USA; c10 Bloomberg School of Public Health, Johns Hopkins University, B615 N Wolfe St, Baltimore, MD 21205, USA

**Keywords:** ***:*** Gender, nutrition, health workers, health systems

## Abstract

A growing body of literature urges policymakers, practitioners and scientists to consider gender in the design and evaluation of health interventions. We report findings from formative research to develop and refine an mHealth maternal nutrition intervention in Nouna, Burkina Faso, one of the world’s most resource-poor settings. Gender was not an initial research focus, but emerged as highly salient during data collection, and thus guided lines of inquiry as the study progressed. We collected data in two stages, first using focus group discussions (FGD; *n* = 8) and later using FGDs (*n* = 2), interviews (*n* = 30) and observations of intervention delivery (*n* = 30). Respondents included pregnant women, breastfeeding mothers and Close-to-Community (CTC) providers, who execute preventative and curative tasks at the community level. We applied Morgan *et al.*’s gender framework to examine intervention content (what a gender-sensitive nutrition programme should entail) and delivery (how a gender-sensitive programme should be administered). Mothers emphasized that although they are often the focus of nutrition interventions, they are not empowered to make nutrition-based decisions that incur costs. They do, however, wield some control over nutrition-related tasks such as farming and cooking. Mothers described how difficult it is to consider only one’s own children during meal preparation (which is communal), and all respondents described how nutrition-related requests can spark marital strife. Many respondents agreed that involving men in nutrition interventions is vital, despite men’s perceived disinterest. CTC providers and others described how social norms and gender roles underpin perceptions of CTC providers and dictate with whom they can speak within homes. Mothers often prefer female CTC providers, but these health workers require spousal permission to work and need to balance professional and domestic demands. We recommend involving male partners in maternal nutrition interventions and engaging and supporting a broader cadre of female CTC providers in Burkina Faso.

**Key Messages**

- Mothers are not empowered to make nutritional changes that involve finances. Male partners are disinterested in nutrition.
- Maternal nutrition interventions should involve male partners to facilitate the implementation of nutritional recommendations and improve communication within couples.
- Social norms suggest that working with female community health workers (CHWs) in the area of maternal nutrition is preferable. Female CHWs have, however, domestic demands and need spousal permission to work.

## Introduction

In recent years, scholars have urged researchers, practitioners and policymakers to evaluate gender in existing health interventions and to design gender-sensitive interventions as a means of increasing effectiveness (Richards *et al.*, 2013; Pratley, 2016; Morgan *et al.*, 2017; Muraya *et al.*, 2017; Steege *et al.*, 2018). While gender-sensitive interventions come in many forms, women’s empowerment is recognized as one of the most promising approaches to gender-sensitive interventions in maternal and child health (Kraft *et al.*, 2014). The rationale behind this is that in low- and middle-income countries women’s empowerment is associated with better health outcomes for mothers and children, including reductions in child mortality (Pratley, 2016; Taukobong *et al.*, 2016). Evidence suggests that empowering women by increasing their access to resources and education enables them to initiate healthcare decisions (Colvin *et al.*, 2013).

In nutrition, particularly, a lack of women’s empowerment has been identified as a key determinant for undernutrition (Bhutta *et al.*, 2008). There is also an association between maternal decision-making power, especially concerning healthcare, and children who are better nourished (Carlson *et al.*, 2015). Increasing women’s income control has been shown to improve children’s nutritional status (Ruel and Alderman, 2013). While pure cash transfers have shown limited positive effects on nutritional status (Van den Bold *et al.*, 2013), agricultural programmes targeting women or focusing on women’s empowerment show better nutritional outcomes for mothers and children (Ruel *et al.*, 2018).

In nutrition, the first 1000 days of life, i.e. from conception to 2 years of age, are recognized as a particularly vital period when maternal nutritional status affects children’s growth and development (Wrottesley *et al.*, 2016). Maternal underweight during pregnancy is a risk factor for preterm labour and low birth weight (Han *et al.*, 2011). Poor female nutritional status is also the main cause of anaemia, globally (Stoltzfus and Dreyfuss, 1998). Iron deficiency anaemia during pregnancy is associated with a higher risk of preterm labour, thus lower birth weight (Allen, 2000), and an increase in pregnancy-related maternal mortality (Brabin *et al.*, 2001). In Africa, micro- and macronutrient-supplementation has been shown to lower mortality and increase birth weight (Wrottesley *et al.*, 2016). However, women’s access to nutritious food is key: in Burkina Faso, a 2-year agricultural programme reduced anaemia and wasting prevalence among children, as well as maternal underweight (Olney *et al.*, 2015, 2016). Women additionally achieved higher empowerment scores (Olney *et al.*, 2016), showing how maternal nutrition and gender go hand in hand.

The goal of this article is to synthesize how gender affects the content and delivery of a nutrition-focused intervention in Burkina Faso, a country where more than half of pregnant women have anaemia (INSD, 2012). While not an a priori focus of the study, the importance of gender emerged early in data collection, and thus informed how the intervention was adapted in terms of content and delivery, which constitute two basic forms of adaptation (Castro *et al.*, 2004), prior to the start of a trial. We first present how gender shapes maternal nutrition at the household level and then how gender shapes Close-to-Community (CTC) providers’ work (CTC providers are those who execute preventative and curative tasks at the community level; Steege *et al.*, 2018). We also chose to focus on CTC providers, since they delivered the nutrition-focused intervention. This article thus builds on and fills a gap in the literature by describing how a maternal nutrition intervention can be designed in a gender-sensitive manner.

## Methods

### Study site and population

Burkina Faso is a landlocked country in Sub-Saharan Africa that ranks 182 of 189 countries in the Human Development Index (United Nations Development Programme, 2019). Income is low at US$731 per person in 2018, placing Burkina Faso among the poorest 10% of countries globally (The World Bank, 2018). Approximately 21% of Burkinabe children under five are malnourished (United Nations Development Programme, 2019), and the country ranks among the lowest 10% of countries globally in terms of gender equity across health, education, economic status and political representation. The average years of education completed are 1.6 years, which is exceptionally low even when compared with other low income, African countries (∼3–6 years)(United Nations Development Programme, 2019). The level of formal education is low for everyone but worse for women: in 2010, 57% of 15- to 19-year-old women had not received any formal education compared with 47% of men (INSD, 2012). Furthermore, educational attainment decreases with age, older women are the least likely to have received any formal education (7% of 50- to 54-year-old women have any formal education) (INSD, 2012). Nearly half of married Burkinabe women (42%) live in polygamy, and 44% of Burkinabe women think that a man has the right to punch his wife (INSD, 2012). A majority (88%) of married women who earn money can decide for themselves how to spend their income (INSD, 2012). Only 8% of Burkinabe women have the principal decision-making power in terms of their own healthcare (INSD, 2012).

Our study site, Nouna town and its surrounding villages, lies in the northwest of Burkina Faso within the Boucle du Mouhon region. About 30 000 people live in Nouna town and about 100 000 people live in the Health and Demographic Surveillance System (HDSS) site, within which we conducted data collection (Sie *et al.*, 2010). In the region, women’s educational level and decision-making power concerning their income are near the country’s average (INSD, 2012). However, more than half of women (56%) living in Boucle du Mouhon accept domestic violence, which is higher than the national average (44%) (INSD, 2012).

### The proposed nutrition intervention

This research stems from a formative study conducted in preparation for a nutrition-promotion trial. We adapted South African maternal nutrition videos that female community health workers (CHWs) originally showed to mothers during home visits via tablets (Rotheram-Borus *et al.*, 2011). The South African research team developed the video intervention using a human-centred design approach grounded on community feedback and iteration (Adam *et al.*, 2019). We chose to follow their approach when adapting the video intervention: to maximize engagement of mothers and CTC providers throughout the adaptation process to ensure internal relevance particularly as several intervention components stem from a different cultural background. The Burkinabe intervention involves CTC providers visiting pregnant and breastfeeding mothers at home to show a set of maternal nutrition videos on a tablet. The videos cover different food groups and emphasize the importance of a varied diet. They feature a mother of a small child making choices about her own and her family’s nutrition regimen (Isler *et al.*, 2020). CTC providers include CHWs, who are predominantly male, and Mentor Mothers (MM) who are exclusively female. CHWs are the government-installed personnel linking a community to its local health centre (Centre de Santé et de Promotion Social, CSPS), and they engage in all areas of prevention. MMs are older women who voluntarily accompany pregnant mothers to health centres, sharing practical advice and sometimes assisting during labour.

### Sample and sampling

Respondents included pregnant and breastfeeding mothers (78 women), CHWs (5 males, 3 females) and MMs (exclusively female). Respondents were purposively selected because they could either receive or deliver the intervention, and they were able to speak and understand Dioula, the most commonly spoken local language. As a means of reflecting the population distribution, we sampled from the catchment areas of two urban and four rural health centres and were guided by CTC providers in terms of identifying pregnant and breastfeeding mothers.

### Qualitative training and data collection

The study consisted of two phases. In the first phase, we gathered data on how to adapt video content. In the second phase, we sought feedback on the adapted videos and preferences for their distribution. Data collectors were female, bilingual (Dioula and French) and came from Nouna town or surrounding villages. They held high school diplomas and had previously conducted research. We worked with only female interviewers to ensure that mothers would be comfortable. We trained data collectors for 3 days on research ethics, maternal nutrition, video interventions, qualitative research and audiotaping techniques. We piloted in-depth interviews (IDIs) and focus group discussions (FGDs) and refined them in close collaboration with the data collection team.

Data collection took place between April and June 2018. We conducted FGDs in quiet areas located in or near health centres. For IDIs and observations, we visited eligible participants at home and delivered the video intervention using tablets. We interviewed 30 mothers and conducted FGDs with 48 mothers, 8 CHWs and 8 MMs. Initially, we intended to work exclusively with CHWs to deliver the video intervention. CHWs are predominantly male; only one health centre in the vicinity employs female CHWs. The reason for this gender imbalance is not clear. However, it became clear that some mothers were uncomfortable interacting with a male CHW. We, therefore, included MMs, and consequently conducted two additional FGDs with MMs and 15 observations of MMs, as well as 15 observations of CHWs. CHWs and MMs were thus involved to a similar extent. For each observation, two research team members joined a CTC provider for a home visit of a pregnant or breastfeeding woman. After initial greetings and explanations, the CTC provider sat with the woman and showed her the videos on a tablet. Research team members sat nearby to observe the CTC provider’s approach to video presentation, the woman’s reactions, how the CTC provider and woman interacted generally, other family members’ involvement and any other pertinent details of the setting in which video viewing took place. After the observation, the mother and CTC provider exchanged thanks and good wishes, the research team extended their own thanks and the team left. For a detailed list of data collection activities see Table 1.

**Table 1:** Data collection activities

Phase	Type of data collection activities	Respondent groups	Number of data collection activities
Phase 1.	Focus group discussions	Mothers	6
		Mentor Mothers (MMs)	1
		Community Health Workers (CHWs)	1
Phase 2.	Observations	Encounters Mothers-MM	15
		Encounters Mothers-CHW	15
	Focus group discussions	MMs	1
		CHWs	1
	In-depth interviews (IDI)	Mothers	30
Total FGDs			10
Total IDIs			30
Total observations			30
Total qualitative data collection activities			70

### Data analysis

We regularly debriefed the data collection team (McMahon and Winch, 2018). Bilingual research assistants transcribed and translated the audio-recorded data from Dioula to French. A member of the research team checked the transcripts for consistency and quality. We developed a codebook grounded on debriefing notes, and structured codes into principal and secondary categories. During initial coding, we refined the codebook and agreed on a final version, which two researchers applied to all transcripts. We incorporated data triangulation by comparing FGDs, IDIs and observations for consistency. Incongruities were discussed with a senior researcher within the study team.

We used existing gender analysis frameworks because they addressed our research questions and we expected them to provide a meaningful basis for our work. We began with the work of Deshmukh and Mechael (2013) because it focuses on gender in mHealth within maternal, newborn and child health. However, this framework was too focused on the intersection of technology and gender to be helpful for analysis of our nutrition-related data. Ultimately, Morgan *et al.* (2016) informed our analysis, because their categorization scheme allowed for a more holistic analysis. Morgan *et al.*’s (2016) framework developed out of a review of existing gender frameworks and argues that gender is a power relation that is negotiated through (1) access to resources, (2) division of labour, (3) social norms and (4) decision-making. Where men are responsible to provide for the family, they are typically favoured in terms of access to resources, both within and beyond the household (March *et al.*, 1999). Types of work are rewarded differently and typical female tasks like household maintenance and childcare are at the lower end of the hierarchy of rewards because they are unpaid and invisible (March *et al.*, 1999). Social norms and rules help to decide in everyday life what behaviour is acceptable, but they can seem set and unchangeable, thus sustaining and justifying gender inequalities (March *et al.*, 1999). As unequal access to resources and a set division of labour are justified by social rules, some individuals gain power over others, thus becoming key decision-makers who can then make decisions that reinforce their own power (March *et al*., 1999). By grouping categories of our codebook as subcategories within the categorization scheme outlined by Morgan *et al.*, we were able to apply this framework to our research process (see Table 2) and content (see Results section). A process of merging, ordering and renaming those subcategories followed. The subcategories presented in the results thus emerged from our own analysis.

**Table 2:** How gender as a power relationship influences research process domains^a^

Domain	Objective response	Accompanying considerations
Who participates as respondents?	Pregnant or breastfeeding mothers Male and female CHWs MMs	We sought to give primarily mothers a platform to express their ideas and included male as well as female CTC providers. Male CHWs additionally gave us their perspective as male partners on the topic.
When and where are data collected?	In the morning and afternoon FGDs in a private area of the health centre IDIs and observations in participants own homes	We planned data collection activities around cooking hours to allow for mothers to fulfil their household duties, and data collection took place in nearby health centres or participants’ own homes to avoid mobility issues.
Who is present?	FGDs included only women except the CHW FGDs Small children were allowed to be present During in-home video viewings, other household members and neighbours were permitted to join	During FGDs with mothers, we intended to have only women present to encourage the mothers to speak. Mothers were told they could bring their small children along if necessary. We conducted gender-mixed FGDs with CHWs, which in retrospect was not ideal.
Who collects data?	Female interviewers from Nouna region	We worked with female interviewers who required more training, but who could more readily encourage female respondents to speak.
Who analyses data?	Two female researchers from Burkina Faso and Germany	We were open to consider gender as it emerged.

^a^ Morgan *et al.* (2016) encourages researchers to ask the following questions to ensure sensitivity to gender throughout the data collection process.

We conducted this research with the approval of the ethics committee of the medical faculty of Heidelberg University (S-140/2018) and the ethics committee of the Burkinabe Health Ministry in Nouna (N°2018-07-/CIE/CRSN). We obtained written consent before all IDIs and FGDs.

## Results

The four aspects that Morgan *et al.*’s (2016) gender framework emphasizes in relation to research content are echoed in our work, as they affect both nutrition at the household level (Section A) and CTC providers’ work (Section B): (1) access to resources (finances, farming/gardening, skills, experience and education); (2) division of labour (who is responsible for nutrition and children); (3) social norms (value of the extended family, mothers’ status, norms regarding contact between male CHWs and mothers); and (4) decision-making (who controls household finances, who decides about female CHWs’ activities). The focus on CTC providers’ work (Section B) emphasizes intervention process; this inclusion is based on an understanding that gender affects who delivers and/or collects information, the strengths and weaknesses of that person in relation to the task at hand, and the manner in which an intervention is introduced, received and perceived, among other things.

### Section A: How gender shapes maternal nutrition at the household level

Respondents consistently emphasized access to resources, especially financial resources and access to farming and gardening, as the most influential factors in terms of guiding decisions about nutrition (Table 3). Social norms, namely how families are comprised, and restrictions that impede mothers’ decision-making power were also described at length, though with less depth. Issues regarding division of labour emphasized male partners’ general disinterest in household nutrition and expectations regarding a mother’s role in meal preparation.

**Table 3:** Morgan *et al.*’s gender analysis framework applied to a nutrition intervention

Areas to examine gender		Answers divided by respondent type		
		Mothers	MMs	CHWs
Access to resources				
(i) Financial resources	• General lack of household finances.	X	X	X
	• Male partners control how much money is provided for nutrition.	X		
	• Mothers have own small income to contribute to the nutrition allowance.	X	X	X
(ii) Farming and gardening	• Households can eat what they plant.	X		X
Social norms				
(i) Value of the extended family	• A need to provide enough food for large, extended families lowers nutrition quality.	X	X	
(ii) Mothers’ status	• Unrealistic expectations of a nutrition allowance or poor meals can spark domestic violence.		X	X
Decision-making (i) Household finances	• Male partners’ support is needed to make nutritional changes.	X		
Division of labour				
(i) Nutrition	• Male partners’ disinterest in nutrition as it is a mother’s domain.	X		X

#### Access to resources

In all FGDs and the vast majority of IDIs, respondents highlighted that financial resources present an extraordinary constraint to varied nutrition. Many mothers underscored the fact that they lack adequate financial resources to implement nutrition recommendations, particularly in relation to buying meat. This can lead to frustration, as expressed by a mother (40 years, 6 children) who reported: ‘We are often told to eat well, but when you don’t have the means it is difficult’ (1. FGD mothers, Nouna).

All respondent groups agreed that both male partners and mothers need to work together to finance household nutrition. Whereas male CHWs emphasized male partners’ challenges to provide adequate household resources, MMs went further and underlined that mothers’ contributions are vital. Many mothers make ends meet by operating small businesses to augment the nutrition allowance. One mother (31 years, 4 children) offered insight into her household’s arrangement: ‘He gives the sorghum and it is up to you to see what you can do to have the sauce (…). So, he struggles to provide for the mush and I (…) will try to obtain the sauce with my small business. We do it like that. One takes the hands and the other takes the feet’ (1. FGD mothers, Toni).

Mothers said that a mother’s relative powerlessness inhibits her from demanding more than the usually insufficient nutrition allowance her male partner provides to acquire cooking ingredients (consisting of grains/sorghum to bargain or money to purchase). All respondents said that most mothers cannot independently decide how much food is in their allowance although a small minority of mothers, those who have direct access to the family granary, can.

Many respondents emphasized that farming is the foundation of household nutrition and would also be a means to bolster it. Farming provides empowerment, as a mother summarized: ‘If we plant it, we can have it’ (IDI D14, Lekuy). Many mothers thought about gardening as a way to provide their family with vegetables. Others sought to achieve dietary variety by cultivating nutrient-rich foods. Mothers did not mention a need for male partner’s approval in relation to farming or gardening. The only barriers mothers talked about were general barriers such as a water supply and migrating animals.

#### Social norms

Social norms, or behavioural expectations at household and community levels, were second only to resource shortages in their power to shape family nutrition profiles. Many respondents described challenges of implementing nutrition recommendations in a context where: (1) large families eat together and (2) conflicts around financing nutrition can spark violence towards mothers.

Regarding household size, mothers emphasized that families are large, include one’s own biological children as well as others’ and additional extended family members. Rather than prioritizing quality, mothers described prioritizing quantity—attempting to ensure that everyone in the extended family receives enough food. Mothers also described the challenges of reconciling their own priorities (giving their children the best food possible) with the reality that co-wives or mothers-in-law may not share this view. ‘You marry, and you live with the whole family of your husband, in the extended family. It is not easy at all. If you want to take care of your household like (the mother in the video who has no co-wives), you will not be able to. But who wouldn’t want to be like her? Who does not want happiness?’ (1. FGD mothers, Nouna)*.* The same social pressures can hinder pregnant or breastfeeding mothers from eating recommended foods, although in some families a pregnancy can justify that a mother receives special food. MMs confirmed that pregnant mothers sometimes eat better quality food than the rest of the family, especially in households with very limited resources.

Limited financial resources are also a common source of contention between spouses, who blame each other for bad nutrition quality. Respondents described how violence towards mothers is considered normal within marital disagreements. CHWs emphasized that mothers who insist on following nutritional recommendations will likely provoke marital tensions, and a mother who places unrealistic demands on her male partner is ‘asking to be beaten’ (2. FGD CHWs, Koussiri). CHWs said, however, that the nutrition videos presented did not nurture unrealistic expectations, but rather encouraged varied nutrition.

#### Decision-making

Respondents agreed that male partners are the primary decision-makers with regards to household finances (including the nutrition allowance) and accepted practices (namely, what mothers should or should not do). Although men are usually uninterested in nutrition, they can unilaterally decide that certain types of food will always be present or absent in the household. Some mothers said that while they appreciated being shown the videos, they nevertheless felt powerless to make nutritional changes without the support of their male partners. Others emphasized that male partners should watch the videos themselves because receiving information second-hand through a wife made it less credible. A mother explained: ‘If he sees it with his own eyes, he will understand’ (IDI D11, Bagala).

#### Division of labour

Division of labour refers to expectations about tasks by gender. Ideally, nutrition interventions would include male partners because they hold the final say in household finances; however, respondents described how health promotion (healthy nutrition and vitamins, breastfeeding and pregnancy) is largely considered a mother’s domain. CHWs said it is, therefore, a perpetual challenge that men, even when explicitly invited, do not usually join their prevention efforts.

### Section B: How gender shapes CTC providers’ work

Respondents emphasized that social norms dictating contact between genders shape male and female CTC providers’ interactions with mothers and their families (Table 4). In less depth, CTC providers described how the influence of the division of labour can provide further challenges for female CTC providers. Specifically, women’s responsibility for household chores and children, and their dependence on spousal permission for their activities, limits their ability to work as CTC providers. Data suggest that gender is less influential than age in determining access to resources such as education and skills (technological proficiency, knowledge transfer, reflection/engagement). Experience in maternal domains (nutrition, childbirth and motherhood) is considered an advantage for female CTC providers.

**Table 4:** Morgan *et al.*’s gender analysis framework for CTC providers

Areas to examine gender		Answers divided by respondent type		
		Mothers	CTC providers	Our observation
Social norms				
(i) Contact between male CHWs and mothers	• CHWs need to prove themselves as people of integrity when dealing with the other gender.		X	
	• Mothers prefer talking with female CTC providers.	X	X	
	• Religious taboos make contact between male CHWs and mothers difficult for some.		X	X
Division of work				
(i) Household chores and children	• Female CHWs are primarily responsible for household chores and children and thus have limited time for CHW-related work.		X	X
Decision-making				
(i) Female CHWs’ activities	• Partners need to approve of female CHWs’ activities.		X	
Access to resources				
(i) Education	• Male and female Community Health Workers (CHW) can read and write.		X	X
	• Mentor Mothers (MM) did not receive formal education and cannot read or write.		X	
(ii) Skills	• Male and female CHWs quickly learn to use a tablet, engage in the intervention and foster knowledge transfer.		X	X
	• MMs struggle with tablet usage, engage less with clients but attract more mothers and children to listen during sensitization.		X	X
(iii) Experience	• Female CTC providers appreciate and adhere to the same gender norms as mothers who thus conclude that they give reasonable advice on cooking and motherhood.	X		X

#### Social norms

Social norms largely determine who will work as a CTC provider, what activities are acceptable for a male versus female CTC provider and how CTC providers are received in communities. CHWs underscored: ‘As a CHW, you need to be a sincere person’ (2. FGD CHWs, Koussiri). They highlighted the need to prove themselves as people of integrity in order to be trusted by the other gender and explained that establishing a strong professional identity was a general prerequisite for community acceptance of interactions across genders. Respondents (including mothers) said that mothers feel embarrassed at the prospect of discussing certain topics with men, including intra-household dynamics around nutrition financing. Some mothers additionally expressed feeling generally more comfortable talking with female CTC providers. In terms of religious taboos, CHWs explained that contact between male CHWs and mothers is difficult for the exceptionally conservative Wahhabi families (Wahhabism is a form of Sunni Islam centred in Saudi Arabia that demands separation of genders in public; Turrittin, 1988; Blanchard, 2007). We observed a male CHW convincing a Wahhabi household head to allow contact with his pregnant daughter-in-law, who was highly uncomfortable while her male partner suspiciously observed the interaction.

#### Division of work

CHWs described how female CHWs’ health-related work directly conflicts with their demands at home, as they are primary caregivers to their children and responsible for household chores, of which male CHWs are generally waived. A CHW explained why he thinks male CHWs can more easily complete their health-related work: ‘Women are busy, they have a lot of work to do. (…) Nobody commands men, they command themselves. They always have time’ (2. FGD CHWs, Koussiri).

#### Decision-making

Respondents agreed that a female CHW needs her male partner’s approval for health-related work. Some male CHWs perceived this as a constant struggle with female CHWs needing to seek a male partner’s permission for each task: ‘She has to go and do a job and her husband will tell her that she is not allowed to leave’ (2. FGD CHWs, Koussiri). Others, male as well as female CHWs, argued that this was more of a one-time issue wherein a female CHW needs her male partner’s permission but can then undertake all duties inherent to the job title: ‘The husband must not get upset. Before the woman was recruited for work, her husband was asked for permission and he agreed’ (2. FGD CHWs, Koussiri). Respondents did not raise this issue in relation to MMs, who are older women, already grandmothers and mothers-in-law, and hold different positions within their households.

#### Access to resources

CHWs (male and female alike) can leverage their education and technological training in order to complete their work, whereas all female CTC providers can access their personal experience as mothers or caretakers for young children. Notably, male and female CHW participants (aged 22–38) had completed several years of formal education, while none of the MMs (aged 50–61) had done so.

The only difference in practices we noted between genders was that male CHWs more readily shared their viewpoints compared with female CHWs, who shared their insights only after engaging with the study team for a longer period. Skills differed between CHWs and MMs, likely depending on education or age. We describe them nevertheless since MMs are in many health centres the only female alternative to working with male CHWs, making those skills consequently a difference between genders, too. In terms of technological proficiency, CHWs (male and female CHWs alike) quickly learned to use a tablet, whereas MMs struggled to use the touchscreen and to remember how to display the videos. During most encounters between MMs and mothers, either the mother took over the manipulation of the tablet or our observation team had to intervene to resolve a technological problem. In terms of fostering knowledge transfer, CHWs created and maintained a calm environment for video viewing, whereas MMs were constantly interrupted by outsiders and curious onlookers during video viewings. An explanation we received is that CHWs are perceived as health workers, whereas MMs are perceived as grandmothers who visit their neighbours. MMs, however, could then promote the message to bigger groups though without the same level of detail. Many MMs enjoyed the conversations that occurred within the context of video viewing as well as the passing on of personal experiences and advice in an informal setting. In terms of reflection and engagement, CHWs engaged with the research team on how to improve the intervention; whereas MMs declined to critique or edit the approach.

Concerning tangible personal experience, some pregnant and breastfeeding mothers told us that they prefer female CTC providers who can leverage personal experience in cooking and motherhood. Mothers can more easily confide in female CTC providers who live in similar circumstances, face similar challenges, and are thus more relatable. Many mothers assured us, however, that they would consider prevention information independently of the CTC providers’ gender.

## Discussion

We analysed our data in relation to (1) intervention content, namely how gender shapes maternal nutrition at the household level; and (2) delivery, namely how gender shapes CTC providers’ work by applying a gender framework that emphasizes four aspects: access to resources, social norms, decision-making and division of labour (Morgan *et al.*, 2016). Our respondents emphasized the importance of access to resources. Mothers did not feel empowered to make nutritional changes that included costs but reported some control over cooking and gardening. Mothers emphasized that meals are prepared for the extended family and food quantity holds priority over nutritional quality. CHWs explained that limited finances lead to nutrition requests often provoking marital disputes. Gender norms also featured in how CTC providers were perceived and who they could talk to. Mothers often preferred female CTC providers, but these providers had to juggle their own domestic demands.

We found Morgan *et al.*’s (2016) framework to be an intuitive and useful tool and the four aspects it underscores in relation to research content proved to be useful main categories to structure our findings. Since the framework developed out of a review of existing gender analysis frameworks and draws on knowledge that has been part of development approaches for decades, this is not surprising. Morgan *et al.*’s (2016) framework makes this knowledge, however, accessible for health system researchers who have no background in gender analysis, which may be its biggest merit. As Morgan *et al.* (2016) emphasize that their framework does not provide a comprehensive list covering all aspects that can arise within the main categories, the framework is highly adaptable across research contexts. We only struggled to classify female CTC provider’s experience bearing and raising children, but ultimately decided experience was a resource, just like educational knowledge, even if it is due to the gendered division of labour. Morgan *et al.* (2016) encourage using the framework within the WHO’s six building blocks of health systems (service delivery, human resources, health financing, leadership/governance, information and research, medical products/technologies) (WHO, 2007). During gender analysis, we found it, however, helpful to distinguish between intervention content, analysing the nutrition situation within households for gender, and intervention delivery, analysing CTC providers’ work for gender. This practical distinction helps to differentiate between the gendered challenges of the target audience and those of health service providers, and this distinction may be helpful for other researchers.

The analysis of our data using Morgan *et al.*’s (2016) framework shows how profoundly gender shapes intra-household nutrition considerations. We found access to resources to be highly influential on nutrition, as reflected in a review that found that increasing women’s power in relation to income control improves children’s nutritional status (Ruel *et al.*, 2013). A more recent review found that nutrition-sensitive agricultural programmes lead to improved nutritional outcomes for mother and child with female empowerment increasing programme effectiveness (Ruel *et al.*, 2018). In Burkina Faso, Helen Keller International conducted a programme that illustrated the impact of women’s access to resources: the 2-year agricultural programme targeted women and integrated empowerment activities, leading to an increase in women’s agricultural production (Olney *et al.*, 2015) and value of their agricultural assets (van den Bold *et al.*, 2015). Ultimately, they reported a reduction in anaemia, diarrhoea and wasting in children aged from 3 to 12.9 months (Olney *et al.*, 2015). Additionally, they found a reduction in mothers’ underweight and increased empowerment measures (Olney *et al.*, 2016).

In this study, we found that mothers are responsible for nutrition and men are generally uninterested in this domain, similar to a Kenyan study (Muraya *et al.*, 2017). That study found, however, that women could autonomously decide to enrol their children in a nutrition intervention (Muraya *et al.*, 2017). The discrepancy between the high decision-making autonomy of women in that study compared with mothers in our own study could have financial roots. Muraya *et al.* do not report any fees charged prior to enrolling children in the intervention (Muraya *et al.*, 2016, 2017) whereas our study was aimed at requiring mothers to alter purchasing patterns in favour of more costly (albeit nutritious) foods. Similar to our findings, Muraya *et al.* emphasize that engaging men in a meaningful manner and bolstering male interest in child nutrition is necessary to improve health outcomes in the long term (Muraya *et al.*, 2017). Other studies also underscored family centeredness (Thuita, 2011). This recommendation is further echoed in a study from rural Gambia, which emphasized that giving women knowledge without the presence or buy-in of partners is insufficient, because men allocate resources and control finances (Mwangome *et al.*, 2010).

Involving men in nutrition interventions is a relatively new topic. However, for health interventions more broadly, there has been a call to educate male partners or household elders as they control household finances and are often gatekeepers to health care (Molyneux *et al.*, 2002; Tolhurst *et al.*, 2008; Colvin *et al.*, 2013; Scott *et al.*, 2014; Osamor and Grady, 2016). In maternal and newborn health, efforts to involve men have been reported, and may provide insights. A recent systematic review found that involving men in maternal and newborn health is associated with increased healthcare-seeking behaviour, better home care practices, improved couples’ communication and more collaborative decision-making (Tokhi *et al.*, 2018). In Burkina Faso itself, involvement of men was also associated with better post-partum practices (Daniele *et al.*, 2018). In relation to joint decision-making and communication, our study identified challenges in the sense that men are expected to provide for their family and women are responsible for nutrition, but both parties suffer when unable to fulfil these expectations or discuss in a collaborative manner. Conflicts about finances have previously been described as a source of conflict, often leading to intimate partner violence (Jewkes, 2002), and low socio-economic status is a risk factor for intimate partner violence against pregnant women across Africa (Shamu *et al.*, 2011). In our study, respondents agreed that integrating male partners into video viewing could facilitate understanding and, echoing the findings of Tokhi *et al.* (2018), mothers desired better communication with their male partners.

Our study found that gender not only affects mothers who receive nutrition interventions, but also female CTC providers who deliver them. Others also found that gender norms influence CTC providers’ work and personal lives (George, 2008; Feldhaus *et al.*, 2015; Steege *et al.*, 2018). In our study, mothers preferred female CTC providers. A Tanzanian study also documented preferences for same-gendered CTC providers to conduct home visits and offer reproductive health counselling (Feldhaus *et al.*, 2015). A 2018 review, however, highlighted the complexity of the situation: while women can be less receptive to treatment uptake when visited by male CTC providers, male CTC providers may be more easily accepted by male decision-makers (Steege *et al.*, 2018). We found that female CTC providers face gendered challenges to engaging in their work, which has again been seen elsewhere (Steege *et al.*, 2018). In Kenya, bearing and caring for children and other household chores was found to be the most important determinant that makes leadership within the health system more difficult for women than men (Muraya *et al.*, 2019). CHWs in our study additionally stressed that a wife needs her male partner’s approval for her professional activities. A lack of intra-household support can hinder female CHWs from joining the CTC workforce (Steege *et al.*, 2018), and engaging in necessary work such as attending trainings (George, 2008). We did not directly hear of a case where a male partner prevented his wife from assuming work as a CHW, although this may be a result of selection bias. We observed that MMs, in contrast to female CHWs, had more freedom to attend intervention activities and did not require spousal permission. Others also report that older women in African societies oftentimes gain freedom, and that sociocultural demands on them are to some extent released (Udvardy and Cattell, 1992). Cumulative life experiences are presumed to endow older women with adequate knowledge to make wise decisions, and some societies do not hinder older women in filling powerful positions such as that of household head.

Nevertheless, gender norms influence how study participants behave during data collection. We noted that male CHWs spoke up more readily than female CHWs. This is consistent with the literature suggesting that women engage less in gender-mixed groups than men (Sell, 1997). A way to address this would be to conduct separate FGDs with female CHWs, as women are known to speak up more readily in all-female groups (Sell, 1997). We also noted that MMs hesitated to critique the intervention. As in rural Burkina Faso men are traditionally responsible for governance and public development (Coulibaly-Lingani *et al.*, 2011), MMs may have felt ill-equipped to critique and change a public intervention.

As explicit recommendations might be helpful for those developing and implementing similar nutrition studies, we provide a Study Implications overview table (see Table 5). A strength of this study was its adaptability; when we noted that gender emerged as important to the study, we integrated those concerns into our study tools and worked to directly examine the issue iteratively. A limitation of this study is that, consequently, we did not initially design the interview guides to investigate gender and failed to investigate the influence of age, comparing, e.g. how younger versus older women’s power varies in relationships. Moreover, we interviewed primarily mothers and we thus lack the perspectives of fathers (although male CHWs could share their perspectives as male partners). Finally, since IDIs and FGDs were conducted in Dioula, transcribed in French and presented here in English, messages could have been comprised or lost during translation.

**Table 5:** Program implications for nutrition studies in similar settings

Research process	Plan data collection activities around cooking hours to allow for mothers to fulfil their household duties. Data collection should take place near participants’ own homes to avoid mobility issues. Separate genders for FGDs (also intervention agents). Allow for mothers to bring along their small children. Pair women with female interviewers.
Intervention agents	Work with female intervention agents. Design the intervention in a way that allows for female intervention agents to complete household duties and manage childcare.
Target group	Focus on foods that are affordable and can be grown easily. Encourage participation of all interested household members in the intervention (e.g. include co-wives, as meal preparation is communal). Include husbands to foster their interest in nutrition and ensure their support (also financially).

## Conclusions

Looking ahead, our study highlights two main opportunities to enhance gender awareness in the design of health and nutrition interventions in settings marked by extreme resource limitations. First, it is essential to involve male partners in maternal nutrition interventions as a means of facilitating the implementation of nutritional advice and fostering constructive couple’s communication. Second, we recommend employing and supporting female CHWs on a wider scale in Burkina Faso, particularly to support priority interventions with women. We also encourage interventionists to incorporate gender-sensitive components in the design and evaluation of health and nutrition interventions. Finally, we hope that our research sparks interest in examining whether and how the inclusion of gender-sensitive components in nutrition interventions impacts women’s empowerment and household nutrition.

## References

[czaa012-B1] Adam M, McMahon SA, Prober C, Bärnighausen T. 2019 Human-centered design of video-based health education: an iterative, collaborative, community-based approach. Journal of Medical Internet Research 21: e12128. 30698531 10.2196/12128 PMC6372941

[czaa012-B2] Allen LH. 2000 Anemia and iron deficiency: effects on pregnancy outcome. The American Journal of Clinical Nutrition 71: 1280S–4S. 10799402 10.1093/ajcn/71.5.1280s

[czaa012-B3] Bhutta ZA, Ahmed T, Black RE et al 2008 What works? Interventions for maternal and child undernutrition and survival. The Lancet 371: 417–40. 10.1016/S0140-6736(07)61693-6 18206226

[czaa012-B4] Blanchard CM. 2007 The Islamic Traditions of Wahhabism and Salafiyya. Washington, DC: Library of Congress, Congressional Research Service.

[czaa012-B5] Brabin BJ, Hakimi M, Pelletier D. 2001 An analysis of anemia and pregnancy-related maternal mortality. The Journal of Nutrition 131: 604S–15S. 11160593 10.1093/jn/131.2.604S

[czaa012-B6] Carlson GJ, Kordas K, Murray‐Kolb LE. 2015 Associations between women’s autonomy and child nutritional status: a review of the literature. Maternal & Child Nutrition 11: 452–82. 24521434 10.1111/mcn.12113 PMC6860340

[czaa012-B7] Castro FG, Barrera M, Martinez CR. 2004 The cultural adaptation of prevention interventions: resolving tensions between fidelity and fit. Prevention Science 5: 41–5. 15058911 10.1023/b:prev.0000013980.12412.cd

[czaa012-B8] Colvin CJ, Smith HJ, Swartz A et al 2013 Understanding careseeking for child illness in sub-Saharan Africa: a systematic review and conceptual framework based on qualitative research of household recognition and response to child diarrhoea, pneumonia and malaria. Social Science & Medicine 86: 66–78. 23608095 10.1016/j.socscimed.2013.02.031

[czaa012-B9] Coulibaly-Lingani P, Savadogo P, Tigabu M, Oden P-C. 2011 Factors influencing people’s participation in the forest management program in Burkina Faso, West Africa. Forest Policy and Economics 13: 292–302.

[czaa012-B10] Daniele MA, Ganaba R, Sarrassat S et al 2018 Involving male partners in maternity care in Burkina Faso: a randomized controlled trial. Bulletin of the World Health Organization 96: 450–61. 29962548 10.2471/BLT.17.206466 PMC6022615

[czaa012-B11] Deshmukh M, Mechael P. 2013 Addressing Gender and Women’s Empowerment in mHealth for MNCH: An Analytical Framework. Washington, DC: mHealth Alliance.

[czaa012-B12] Feldhaus I, Silverman M, LeFevre AE et al 2015 Equally able, but unequally accepted: gender differentials and experiences of community health volunteers promoting maternal, newborn, and child health in Morogoro Region, Tanzania. International Journal for Equity in Health 14: 70. 26303909 10.1186/s12939-015-0201-z PMC4548695

[czaa012-B13] George A. 2008 Nurses, community health workers, and home carers: gendered human resources compensating for skewed health systems. Global Public Health 3: 75–89. 19288344 10.1080/17441690801892240

[czaa012-B14] Han Z, Mulla S, Beyene J, Liao G, McDonald SD; Knowledge Synthesis Group. 2011 Maternal underweight and the risk of preterm birth and low birth weight: a systematic review and meta-analyses. International Journal of Epidemiology 40: 65–101. 21097954 10.1093/ije/dyq195

[czaa012-B15] INSD. 2012 Enquête Démographique et de Santé et à Indicateurs Multiples du Burkina Faso 2010. Calverton, MD: Institut National de la Statistique et de la Démographie (INSD) and ICF International.

[czaa012-B16] Isler J, Sawadogo NH, Harling G, et al. 2020. Iterative adaptation of a mobile nutrition video-based intervention across countries using human-centered design: qualitative study. *JMIR mHealth and uHealth*, **8**: e17666. 10.2196/17666 PMC7351258 32012112

[czaa012-B17] Jewkes R. 2002 Intimate partner violence: causes and prevention. The Lancet 359: 1423–9. 10.1016/S0140-6736(02)08357-5 11978358

[czaa012-B18] Kraft JM, Wilkins KG, Morales GJ, Widyono M, Middlestadt SE. 2014 An evidence review of gender-integrated interventions in reproductive and maternal-child health. Journal of Health Communication 19: 122–41. 25207450 10.1080/10810730.2014.918216 PMC4205884

[czaa012-B2882331] March C, Smyth I, Mukhopadhyay M. 1999. *A Guide to Gender-Analysis Frameworks*. Oxfam.

[czaa012-B19] McMahon SA, Winch PJ. 2018 Systematic debriefing after qualitative encounters: an essential analysis step in applied qualitative research. BMJ Global Health 3: e000837. 10.1136/bmjgh-2018-000837 PMC6135453 30233833

[czaa012-B20] Molyneux CS, Murira G, Masha J, Snow R. 2002 Intra-household relations and treatment decision-making for childhood illness: a Kenyan case study. Journal of Biosocial Science 34: 109–31. 11814209

[czaa012-B21] Morgan R, George A, Ssali S et al 2016 How to do (or not to do)… gender analysis in health systems research. Health Policy and Planning 31: 1069–78. 27117482 10.1093/heapol/czw037 PMC6616028

[czaa012-B22] Morgan R, Tetui M, Muhumuza Kananura R, Ekirapa-Kiracho E, George A. 2017 Gender dynamics affecting maternal health and health care access and use in Uganda. Health Policy and Planning 32: v13–21. 29244103 10.1093/heapol/czx011 PMC5886085

[czaa012-B23] Muraya KW, Govender V, Mbachu C, Uguru NP, Molyneux S. 2019 ‘Gender is not even a side issue… it’s a non-issue’: career trajectories and experiences from the perspective of male and female healthcare managers in Kenya. Health Policy and Planning 34: 249–56. 31329845 10.1093/heapol/czz019 PMC6661539

[czaa012-B24] Muraya KW, Jones C, Berkley JA, Molyneux S. 2016 Perceptions of childhood undernutrition among rural households on the Kenyan coast—a qualitative study. BMC Public Health 16: 693. 27484493 10.1186/s12889-016-3157-z PMC4971694

[czaa012-B25] Muraya KW, Jones C, Berkley JA, Molyneux S. 2017 “If it’s issues to do with nutrition… I can decide…”: gendered decision-making in joining community-based child nutrition interventions within rural coastal Kenya. Health Policy and Planning 32: v31–9. 29244104 10.1093/heapol/czx032 PMC5886246

[czaa012-B26] Mwangome M, Prentice A, Plugge E, Nweneka C. 2010 Determinants of appropriate child health and nutrition practices among women in rural Gambia. Journal of Health, Population, and Nutrition 28: 167–72. 10.3329/jhpn.v28i2.4887 PMC2980879 20411680

[czaa012-B27] Olney DK, Bliznashka L, Pedehombga A et al 2016 A 2-year integrated agriculture and nutrition program targeted to mothers of young children in Burkina Faso reduces underweight among mothers and increases their empowerment: a cluster-randomized controlled trial. The Journal of Nutrition 146: 1109–17. 27075910 10.3945/jn.115.224261

[czaa012-B28] Olney DK, Pedehombga A, Ruel MT, Dillon A. 2015 A 2-year integrated agriculture and nutrition and health behavior change communication program targeted to women in burkina faso reduces anemia, wasting, and diarrhea in children 3–12.9 months of age at baseline: a cluster-randomized controlled trial–3. The Journal of Nutrition 145: 1317–24. 25904734 10.3945/jn.114.203539

[czaa012-B29] Osamor PE, Grady C. 2016 Women’s autonomy in health care decision-making in developing countries: a synthesis of the literature. International Journal of Women’s Health 8: 191. 10.2147/IJWH.S105483 PMC4908934 27354830

[czaa012-B30] Pratley P. 2016 Associations between quantitative measures of women’s empowerment and access to care and health status for mothers and their children: a systematic review of evidence from the developing world. Social Science & Medicine 169: 119–31. 27716549 10.1016/j.socscimed.2016.08.001

[czaa012-B31] Richards E, Theobald S, George A et al 2013 Going beyond the surface: gendered intra-household bargaining as a social determinant of child health and nutrition in low and middle income countries. Social Science & Medicine 95: 24–33. 22809796 10.1016/j.socscimed.2012.06.015

[czaa012-B32] Rotheram-Borus MJ, Le Roux IM, Tomlinson M et al 2011 Philani plus (+): a mentor mother community health worker home visiting program to improve maternal and infants’ outcomes. Prevention Science 12: 372–88. 21850488 10.1007/s11121-011-0238-1 PMC3907085

[czaa012-B33] Ruel MT, Alderman HMaternal and Child Nutrition Study Group 2013 Nutrition-sensitive interventions and programmes: how can they help to accelerate progress in improving maternal and child nutrition? The Lancet 382: 536–51. 10.1016/S0140-6736(13)60843-0 23746780

[czaa012-B34] Ruel MT, Quisumbing AR, Balagamwala M. 2018 Nutrition-sensitive agriculture: what have we learned so far? Global Food Security 17: 128–53.

[czaa012-B35] Scott K, McMahon S, Yumkella F, Diaz T, George A. 2014 Navigating multiple options and social relationships in plural health systems: a qualitative study exploring healthcare seeking for sick children in Sierra Leone. Health Policy and Planning 29: 292–301. 23535712 10.1093/heapol/czt016

[czaa012-B36] Sell J. 1997 Gender, strategies, and contributions to public goods. Social Psychology Quarterly 60: 252.

[czaa012-B37] Shamu S, Abrahams N, Temmerman M, Musekiwa A, Zarowsky C. 2011 A systematic review of African studies on intimate partner violence against pregnant women: prevalence and risk factors. PLoS One 6: e17591. 21408120 10.1371/journal.pone.0017591 PMC3050907

[czaa012-B38] Sie A, Louis V, Gbangou A et al 2010 The health and demographic surveillance system (HDSS) in Nouna, Burkina Faso, 1993–2007. Global Health Action 3: 5284. 10.3402/gha.v3i0.5284 PMC2940452 20847837

[czaa012-B39] Steege R, Taegtmeyer M, McCollum R et al 2018 How do gender relations affect the working lives of close to community health service providers? Empirical research, a review and conceptual framework. Social Science & Medicine 209: 1–13. 29777956 10.1016/j.socscimed.2018.05.002

[czaa012-B40] Stoltzfus RJ, Dreyfuss ML. 1998 Guidelines for the Use of Iron Supplements to Prevent and Treat Iron Deficiency Anemia. Washington, DC: ILSI Press.

[czaa012-B41] Taukobong HFG, Kincaid MM, Levy JK et al 2016 Does addressing gender inequalities and empowering women and girls improve health and development programme outcomes? Health Policy and Planning 31: 1492–514. 27371549 10.1093/heapol/czw074

[czaa012-B42] The World Bank. 2018 World Bank national accounts data and OECD National Accounts data files. https://www.macrotrends.net/countries/BFA/burkina-faso/gdp-per-capita, accessed 10 February 2020.

[czaa012-B43] Thuita F. 2011 Engaging Grandmothers and Men in Infant and Young Child Feeding and Maternal Nutrition: Report of a Formative Assessment in Eastern and Western Kenya. Kenya: Ministry of Health Kenya-USAID’s Infant and Young Child Nutrition Project.

[czaa012-B44] Tokhi M, Comrie-Thomson L, Davis J et al 2018 Involving men to improve maternal and newborn health: a systematic review of the effectiveness of interventions. PLoS One 13: e0191620. 29370258 10.1371/journal.pone.0191620 PMC5784936

[czaa012-B45] Tolhurst R, Amekudzi YP, Nyonator FK, Squire SB, Theobald S. 2008 “He will ask why the child gets sick so often”: the gendered dynamics of intra-household bargaining over healthcare for children with fever in the Volta Region of Ghana. Social Science & Medicine 66: 1106–17. 18166257 10.1016/j.socscimed.2007.11.032

[czaa012-B46] Turrittin J. 1988 Men, women, and market trade in rural Mali, West Africa. Canadian Journal of African Studies 22: 583–604.

[czaa012-B47] Udvardy M, Cattell M. 1992 Gender, aging and power in sub-Saharan Africa: challenges and puzzles. Journal of Cross-Cultural Gerontology 7: 275–88. 24389691 10.1007/BF01848695

[czaa012-B48] United Nations Development Programme. 2019 Human Development Reports. http://hdr.undp.org/en/countries/profiles/BFA, accessed 10 February 2020.

[czaa012-B49] van den Bold M, Dillon A, Olney D et al 2015 Can integrated agriculture-nutrition programmes change gender norms on land and asset ownership? Evidence from Burkina Faso. The Journal of Development Studies 51: 1155–74. 30363952 10.1080/00220388.2015.1036036 PMC6183935

[czaa012-B50] Van den Bold M, Quisumbing AR, Gillespie S. 2013 *Women's Empowerment and Nutrition: An Evidence Review* Washington, DC: International Food Policy Research Institute.

[czaa012-B51] WHO. 2007 *Everybody’s Business: Strengthening Health Systems to Improve Health Outcomes: WHO’s Framework for Action*. Geneva: World Health Organization.

[czaa012-B52] Wrottesley S, Lamper C, Pisa P. 2016 Review of the importance of nutrition during the first 1000 days: maternal nutritional status and its associations with fetal growth and birth, neonatal and infant outcomes among African women. Journal of Developmental Origins of Health and Disease 7: 144–62. 26279311 10.1017/S2040174415001439

